# DACH1 inhibits the proliferation and invasion of lung adenocarcinoma through the downregulation of peroxiredoxin 3

**DOI:** 10.1007/s13277-016-4811-x

**Published:** 2016-01-25

**Authors:** Ji Zhu, Cong Wu, Huafei Li, Yang Yuan, Xiaotian Wang, Tiejun Zhao, Jibin Xu

**Affiliations:** 1 Department of Cardiothoracic Surgery, Changhai Hospital Affiliated to the Second Military Medical University, 168 Changhai Road, 200433 Shanghai, People’s Republic of China; 2 Department of Laboratory Diagnosis, Changhai Hospital Affiliated to the Second Military Medical University, Shanghai, People’s Republic of China; 3 International Joint Cancer Institute, Translational Medicine Research Institute, The Second Military Medical University, Shanghai, China; 4 Department of Cardiothoracic Surgery, Changzheng Hospital Affiliated to the Second Military Medical University, 415 Fengyang Road, 200433 Shanghai, People’s Republic of China

**Keywords:** DACH1, Lung adenocarcinoma, Cell cycle, PRX3

## Abstract

In this study, we found the expression of Dachshund 1 (DACH1) is downregulated while peroxiredoxin 3 (PRX3) upregulated in both lung adenocarcinoma tissues and cells. Transfection of DACH1 can significantly downregulate PRX3 expression in targeting lung adenocarcinoma cells. Further experimental results demonstrated the evidence that overexpression of DACH1 resulted in significant retardation of in vitro proliferation and invasion of lung adenocarcinoma cells. Direct upregulation of PRX3 by co-transfection of PRX3 messenger RNA (mRNA) can prevent the above alteration caused by DACH1 transfection. Besides, lower DACH1 expression significantly correlated with tumor diameter and tumor invasion in all the 36 patients diagnosed with lung adenocarcinoma in our hospital during the past months. In conclusion, DACH1 can inhibit the proliferation and invasion of lung adenocarcinoma through the downregulation of PRX3. Decreased expression of DACH1 is involved in the initiation and development of lung cancer, which might be an adverse prognostic factor of lung adenocarcinoma.

**Electronic supplementary material:**

The online version of this article (doi:10.1007/s13277-016-4811-x) contains supplementary material, which is available to authorized users.

## Introduction

Lung cancer is the most common cancer in men worldwide with an age-standardized rate (ASR) of 33.8 per 100,000 and the fourth most frequent cancer in women (13.5 per 100,000) [1, 2]. Lung cancer is the leading cause of cancer-related mortality among both men and women worldwide with approximately 160,000 deaths reported each year in the USA [3, 4]. Non-small cell lung cancer (NSCLC) constitutes approximately 80 % of all lung cancers and includes adenocarcinoma and squamous cell carcinoma, which are histologically distinct from small cell lung cancer [5]. Although significant advances have been made with conventional therapies, including surgery, chemotherapy, and radiotherapy, the poor prognosis and low overall survival (OS) rate highlights the urgent need for developing novel therapeutic methods [6]. Recently, patients with activating mutations of the epidermal growth factor receptor (EGFR) gene have shown an effective response to small-molecule competitive inhibitors of the EGFR tyrosine kinase such as gefitinib (Iressa, AstraZeneca) and erlotinib (Tarceva, Roche) [7]. However, these drugs appear to be less effective in patients without EGFR mutation [8, 9]. Besides, patients rapidly develop drug resistance, which typically occurs 8–12 months from the start of treatment [10]. Thus, the identification of novel targets is of great importance for curing lung cancers.

The *Dachshund* gene (*dach*) was originally cloned in *Drosophila* as a dominant inhibitor of *ellipse* and encodes a key component of the retinal determination gene network (RDGN) [11]. The mammalian Dachshund 1 (DACH1) regulates the expression of target genes, in part, through interacting with DNA-binding transcription factors (*c-Jun*, *Smads*, *Six*, and *ERα*), and in part through intrinsic DNA sequence-specific binding properties via Forkhead binding sequences [11–13]. DACH1 is expressed widely in normal adult tissues, which functions as a tumor suppressor in a variety of malignant tumors in previous studies. Wu K et al’s studies demonstrated that DACH1 blocks mammary tumor growth through downregulating *Nanog* and *Sox2*, and reduced DACH1 protein levels correlated significantly with poor prognosis of breast cancer [14, 15]. Y. Yamada et al. revealed that lower DACH1 level correlates with a poor prognosis of gastric cancer patients [16]. DACH1 level is also found decreased in prostate cancer, and re-introduction of DACH1 inhibits prostate cancer cell proliferation in vitro [13]. Recently, several studies focused on the effects of DACH1 on the proliferation and invasion of lung cancer cells. Ke Chen et al. identified DACH1 as a novel p53 binding partner that participates in p53-mediated induction of p21^CIP1^ and cell cycle arrest [12]. Na Han et al’s study revealed low expressions of DACH1 predicted unfavorable prognosis for survival, and CXCL5 was identified as a downstream target of DACH1-mediated repression of cell invasion and tumorigenesis [17]. While in this study, we identified that peroxiredoxin 3 (PRX3) is a key target of DACH1.

PRX3 is a member of the peroxiredoxin family, which contains a conserved N-terminal and participates in degradation of H_2_O_2_ [18]. PRX3 is specifically located in the mitochondria, which exhibits antioxidant properties, regulates cell signaling and proliferation, protects protein structure, and inhibits redox-dependent pathways of apoptosis activation, acting as an important role in cell protection from oxidative stress [19, 20]. In the past decades, more and more researches demonstrated that PRX3 take parts in the development and progression of malignant tumors. Jinxia Hu et al. revealed that increased expression of PRX3 in cervical cancer is a potential marker for cell proliferation [21]. Nonn et al. reported that PRX3 played a protective role against drug-induced oxidative stress and subsequent apoptosis of thymoma cells [22]. Kalinina et al. revealed that an increase in the expression of PRX3 was detected in cisplatin-resistant cancer cells, including a cisplatin-resistant breast cancer cell line (MCF-7), a cisplatin-resistant human erythroleukemia cell line (K562), and a cisplatin-resistant human ovarian carcinoma cell line (SKOV-3), while this increased expression protects cells from apoptosis and participates in the regulation of cell proliferation [23].

In this study, we explored the aberrant status, cancer-related functions, and potential tumor suppressing mechanisms of DACH1 in lung adenocarcinoma tissues and cell lines. Our results suggest that DACH1 acts as a tumor suppressor by targeting PRX3 functional target genes in malignant lung cells. More important, lower DACH1 expression significantly correlated with tumor diameter and tumor invasion. All these results demonstrated that DACH1 might be an efficient tumor suppressor in lung adenocarcinoma tumor, which merits further investigation in the clinic.

## Materials and methods

### Patients and specimens

Fresh-frozen human lung adenocarcinoma tissues (*n* = 36) and matched distant normal lung tissues were obtained from patients who underwent radical surgery between 7 April and 12 August, 2014, at Changhai Hospital affiliated to the Second Military Medical University (Shanghai China) with informed consent. The patients included 21 males and 15 females, with an average age of 58 years. None of these patients received radiotherapy or chemotherapy before surgical resection. All 36 cases were reviewed for histological subtype, differentiation and tumor stage. The histological diagnosis and grade were evaluated on hematoxylin and eosin-stained sections according to the World Health Organization (WHO) guidelines of classification. ACCP classification was used to classify specimens as stages I (*n* = 8), II (*n* = 11), and IIIa (*n* = 17) [24].

### Cell culture

Two human lung adenocarcinoma cell lines (LTEP-α-2 and A549) and a normal alveolar epithelium cell line (BEAS-2B) were obtained from the American Type Culture Collection (ATCC). Cells were cultured in Dulbecco’s Modified Eagle’s Medium (DMEM) (Gibco, Carlsbad, USA) supplemented with 10 % bovine calf serum (Gibco, Carlsbad, USA) and maintained at 37 °C in an atmosphere of humidified air with 5 % CO_2_.

### Immunohistochemistry

Immunohistochemical (IHC) analysis of human lung adenocarcinoma tissues and adjacent non-tumor tissues was conducted using monoclonal DACH1 or PRX3 antibodies (Cell Signaling Technology, USA, 1:1000 dilution) as in previous descriptions [13]. Immunohistochemical staining scores were evaluated as the percentage of positive cells and counted by two independent persons not aware of the patient information.

### Western blotting

Whole cell/tissue lysates were harvested in ice-cold lysis buffer (10 mM Tris–HCl, 1 mM EDTA, 0.1 % Triton X-100 and 0.1 % SDS, pH = 7.4) containing protease inhibitors (2 μg/mL aprotinin, 10 μg/mL antipain, 2 μg/mL pepstatin, and 2 mM benzamide). After the removal of cell debris by centrifugation (12,000*g* × 10 min), the protein concentration in the supernatants was measured using bicinchoninic acid protein assay reagent (Pierce Chemical Co., Rockford, IL, USA) according to the product information. Ten micrograms of total protein were subjected to SDS-PAGE and immunoblotted with DACH1/PRX3 antibodies (Cell Signaling Technology, USA). GAPDH (glyceraldehyde-3-phosphate dehydrogenase) was used as a reference protein to analyze the DACH1 quantitatively [25].

### Real-time PCR

Total RNA was reverse transcribed into complementary DNA (cDNA) by using AMV Reverse Transcriptase (Takara, Japan) with oligo (dT) 18 or specific RT primer. Equal amounts of the cDNA products were used as templates for subsequent PCR amplification using the q-PCR thermal cycler (steponeplus, ABI, USA). The oligonucleotide primer sequences used in this research were illustrated in Supplementary Table 1.

### Plasmid transfection

Cells were grown till 70–80 % confluence and respectively transfected with vector (pcDNA), exogenously DACH1 expression plasmid (DACH1 pcDNA), and PRX3 expression plasmid (PRX3 pcDNA) using Lipofectamine 2000 (Invitrogen Life Technologies, Carlsbad, CA, USA) according to the manufacturer’s instructions, respectively. After 48 h, cells were harvested, followed by limited dilution in a 96-well plate for the generation of individual clones. Two weeks later, cells were harvested for further experiments.

### Dual-luciferase report assay

Dual-luciferase assay was performed as in previous description [26]. Briefly, the 3’UTR of the PRX3 gene was cloned into the pGL3-basic vector to generate a PRX3-pGL3 vector. Harvest cells were plated into a 24-well plate at approximately 80 % confluence, then co-transfected with DACH1 and PRX3 expressed vectors using lipofectamine 2000 transfection agents according to the manufacturer’s protocol. Subsequent to 24 h of transfection, the cells were harvested, lysed, and analyzed using the Dual-Luciferase Reporter Assay System kit (Promega, USA). All the experiments were performed in triplicate.

### Cell cycle analysis

Harvested cells were re-suspended with 50 μg/ml propidium iodide (PI, Sigma-Aldrich) for 30 min in the dark before analysis. The percentages of cells in different phases of cell cycle were determined using a FACSCalibur Flow Cytometer with Cell Quest 3.0 software (BD Biosciences). Experiments were performed in triplicate.

### Colony formation assay

Harvested cells were seeded into 6-cm cell culture dishes (500 per dish) and incubated at 37 °C for 2 weeks. Colonies were visualized by 0.04 % crystal violet staining, and colonies more than 50 μm in diameter were counted using an Omnicon 3600 image analysis system.

### Cell proliferation assays

Cell proliferation assays were performed according to previous descriptions [27]. Briefly, cells were seeded into 96-well tissue culture plates at an initial density of 3000 cells/well in 100 μl of culture medium. After different time intervals, the cell viability was determined with a Cell Counting Kit-8 (CCK-8, Dojindo Molecular Technologies, Oslo, Norway) following the manufacturer’s protocol.

### Statistical analysis

SPSS (version 16.0) for windows was used for all statistical analyses. The chi-squared test was used to examine possible correlations between DACH1 expression and clinicopathologic factors. Pearson’s correlation coefficient statistics method was used to examine the correlations between levels of DACH1 expression and target gene expression. *P* < 0.05 was considered statistically significant.

## Results

### Reduced DACH1 expression in both lung adenocarcinoma tissues and cells

**Fig. 1:**
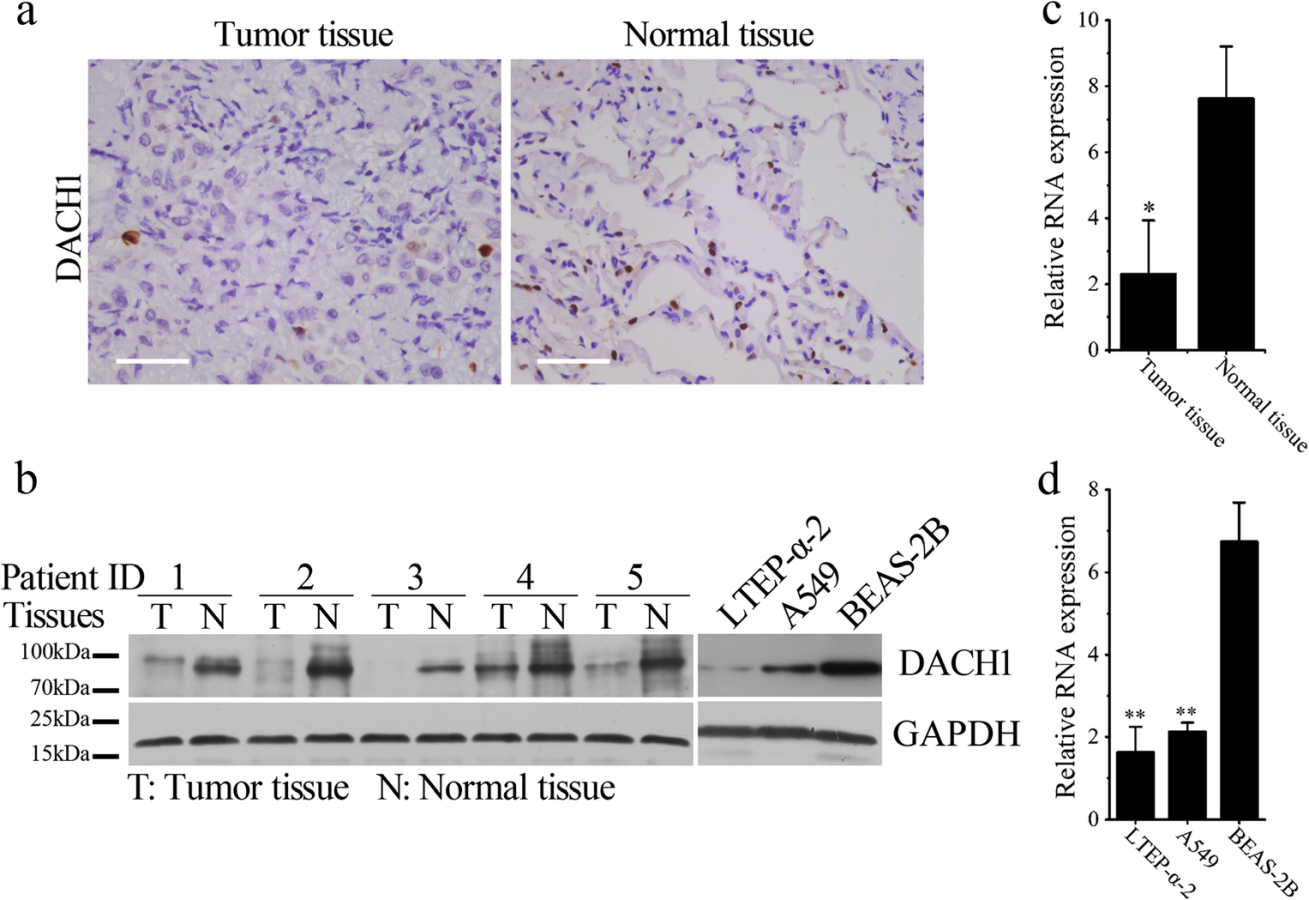
Decreased DACH1 expression in lung cancer tissues and cells. **a** Representative photos of immunohistochemistry for DACH1 in pairs of lung cancer tissues and adjacent non-tumor tissues (*scale bar* 50 μm). **b** *Left panel*: representative photos of Western blotting for DACH1 in pairs of lung cancer tissues and adjacent non-tumor tissues. *Right panel*: Western blotting analysis for DACH1 expression in LTEP-α-2, A549, and BEAS-2B cells. **c**–**d** Relative levels of DACH1 in normal and malignant tissues (**c**) and cells (**d**) by real-time PCR analysis. Data are shown as mean ± SEM, *p < 0.05, **p < 0.01

In order to determine the alteration of DACH1 expression in lung cancer, we performed Western blotting (WB) and IHC analysis of DACH1 levels between the 36 pairs of lung adenocarcinoma tissues and adjacent non-tumor tissues. Figure 1a, b respectively demonstrates the representative IHC and WB photos, from which we can see that DACH1, which confined to the nuclear of normal and malignant pulmonary epithelial cells, is obviously downregulated in lung adenocarcinoma tissues. Consistent results were obtained in cultured cell lines by WB with the results shown in the right panel of Fig. 1b, which reveals that comparing with BEAS-2B cells (a normal alveolar epithelium cell line), DACH1 expression was significantly decreased in both two lung adenocarcinoma cell lines, LTEP-α-2 and A549. The relatively low expression of DACH1 in lung adenocarcinoma tissues and cells was validated by real-time PCR (RT-PCR) analysis, with results showing in Fig. 1c, d (*p < 0.05, **p < 0.01).

### PRX3 was significantly upregulated in both lung adenocarcinoma tissues and cells

**Fig. 2:**
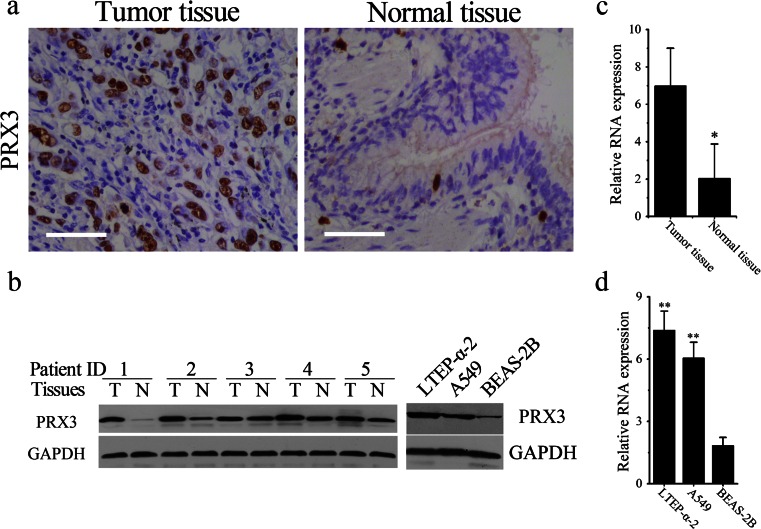
Increased PRX3 expression in lung cancer tissues and cells. **a** Representative photos of immunohistochemistry for PRX3 in pairs of lung cancer tissues and adjacent non-tumor tissues (*scale bar* 50 μm). **b** *Left panel*: representative photos of Western blotting for PRX3 in pairs of lung cancer tissues and adjacent non-tumor tissues. *Right panel*: Western blotting analysis for PRX3 expression in LTEP-α-2, A549, and BEAS-2B cells. **c**–**d** Relative levels of PRX3 in normal and malignant tissues (**c**) and cells (**d**) by real-time PCR analysis. Data are shown as mean ± SEM, *p < 0.05, **p < 0.01

Similar experiments were also employed to compare the expression of PRX3 in normal and malignant lung tissues and cells. Figure 2a, b respectively demonstrates the representative IHC and WB photos, from which we can see that the expression of PRX3, which confined to the cytoplasm of normal and malignant pulmonary epithelial cells, is significantly increased in lung adenocarcinoma tissues and cells. Also, RT-PCR was used to compare the messenger RNA (mRNA) levels of PRX3 in normal and malignant lung epithelial tissues and cells. Figure 2c demonstrates that the average level of PRX3 mRNA was significantly decreased in all the 36 collected lung adenocarcinoma tissues (*p < 0.05) in comparison with normal tissues. In accordance, the levels of PRX3 in both LTEP-α-2 and A549 cells were remarkably higher than that in BEAS-2B cells (**P < 0.01, Fig. 2d).

### Overexpression of DACH1 downregulates PRX3 levels in lung cancer cells

**Fig. 3:**
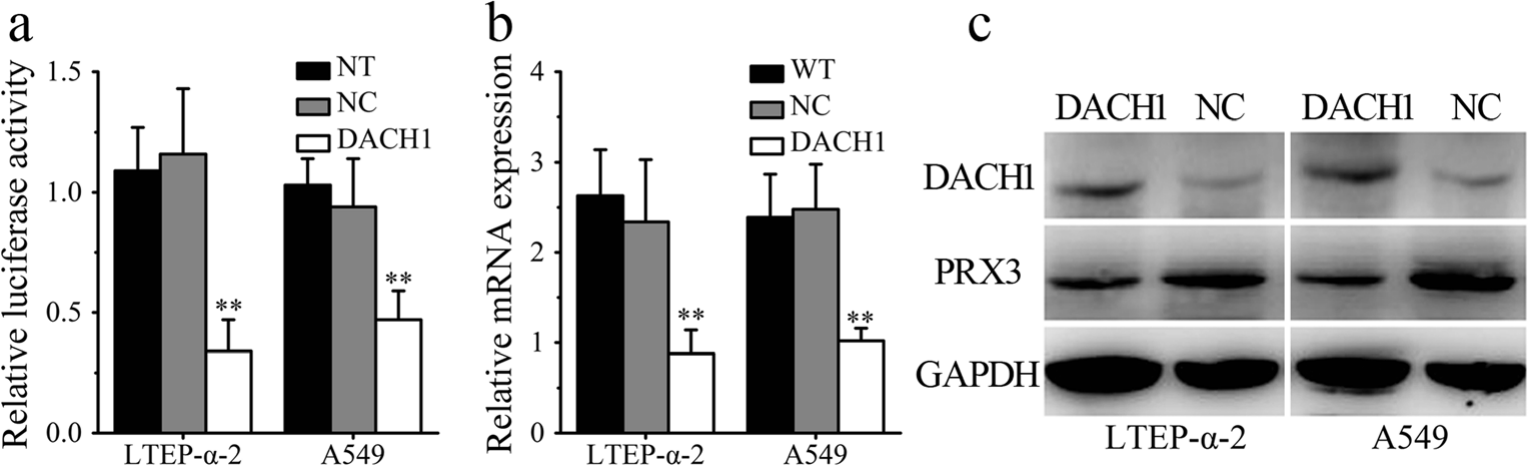
PRX3 may be a direct target of DACH1 in vitro. **a** The luciferase activity was significantly lower when transfected with DACH1 but not empty vectors in both LTEP-α-2 and A549 cells. **b** Transfection with DACH1 can remarkably downregulate cellular PRX3 expression in both LTEP-α-2 and A549 cells by real-time PCR analysis. **c** Western blotting analysis for DACH1 and PRX3 expression in wild-type and DACH1 transfected lung cancer cells (LTEP-α-2 and A549). Data are shown as mean ± SEM (*n* = 3), **p < 0.01

Considering the downregulation of DACH1 (Fig. 1) and upregulation of PRX3 (Fig. 2) expression in human lung adenocarcinoma cells and tissues, we hypothesized that there may be a negative correlation between their intracellular levels. In order to verify this hypothesis, the luciferase reporter system was employed to investigate the regulatory role of DACH1 in the expression of PRX3. Figure 3a revealed that transfection of DACH1 but not empty vectors significantly decreased the luciferase levels expressed in both lung cancer cells (**P < 0.01). The results were confirmed by direct detection of PRX3 levels by RT-PCR and WB analysis. As we can see from Fig. 3b–c, the intracellular levels of PRX3 in both mRNA and protein levels experienced a significant decrease in DACH1-transfected cancer cells (**P < 0.01).

### Overexpression of DACH1 inhibits cell proliferation and migration of lung adenocarcinoma cells

**Fig. 4:**
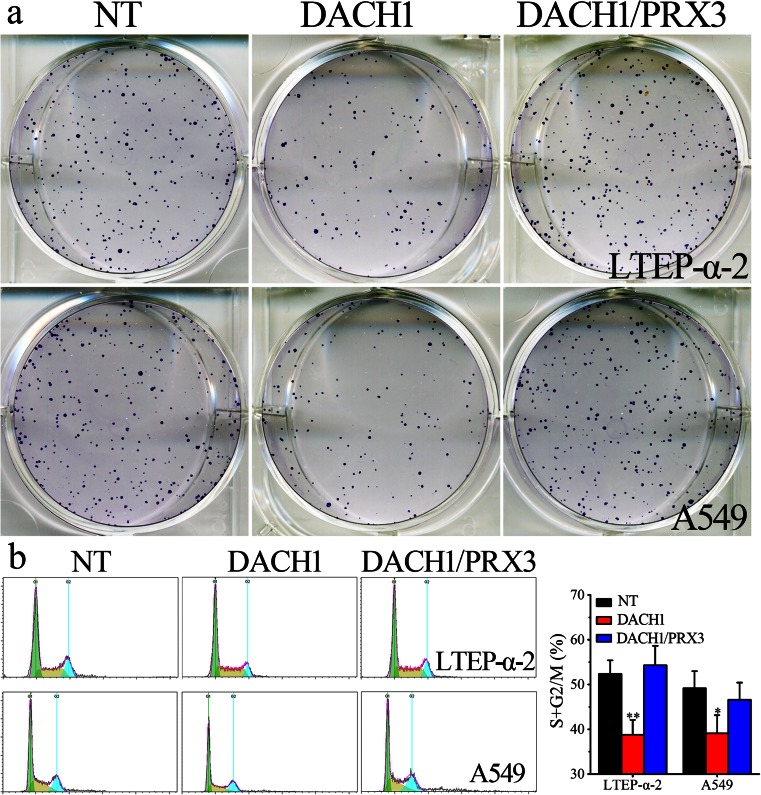
The influence of PRX3 and DACH1 on migration ability and cell cycle arrest of lung cancer cells. **a** Colony-forming assays comparing wild-type lung cancer cells and cells transfected with DACH1 and PRX3. **b** Cell cycle distribution analysis of both LTEP-α-2 and A549 cells transfected with DACH1 and PRX3 in comparison with wild-type cells by flow cytometry

**Fig. 5:**
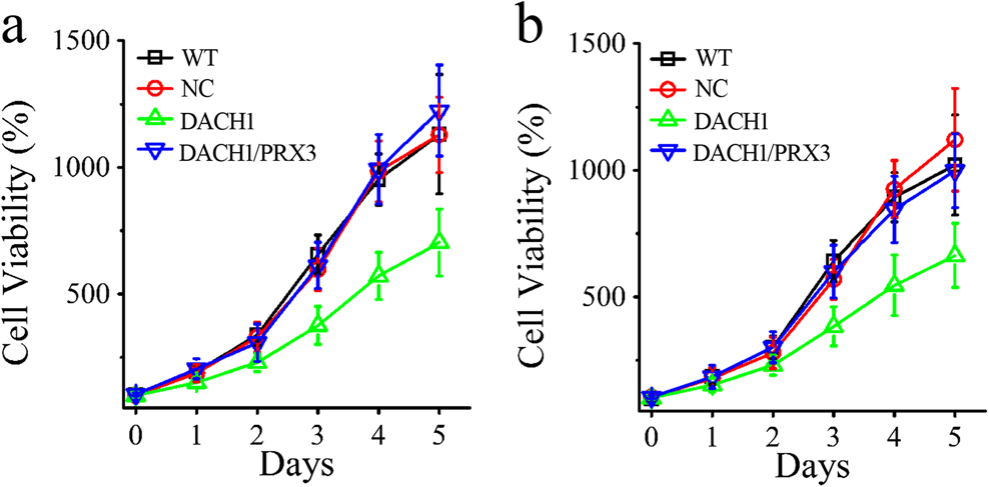
The influence of PRX3 and DACH1 on proliferation of lung cancer cells. **a** Viability of wild-type LTEP-α-2 cells and cells infected with DACH1 with or without PRX3 by CCK-8 assays. **b** Viability of wild-type A549 cells and cells infected with DACH1 with or without PRX3 by CCK-8 assays. Data are shown as mean ± SD, *p < 0.05, **p < 0.01

In order to investigate the influence of DACH1 on cell proliferation and invasion, wild-type lung cancer cells and cells infected with DACH1 or empty vectors (NC) were employed in the subsequent studies. The colony formation assay revealed that the counts of colonies formed by DACH1-transfected lung adenocarcinoma cells were much less than those of WT cells (Fig. 4a). Figure 4b demonstrates that both lung adenocarcinoma cells were enriched at S and G2/M phases when DACH1 was overexpressed (**P < 0.01). Similar results were obtained in cell proliferation assays, the results of which (Fig. 5a) reveals that subsequent to a 5-day-period, cell proliferation ability was lower in DACH1 transfection groups than in the non-transfection (WT) and mock-vehicle groups (NC). The remarkable decrease was firstly observed on day 2. It should be noted here that the negative regulating roles of DACH1 on cell proliferation and invasion can be significantly prevented by co-transfection of PRX3 (Figs. 4 and 5). All these results clearly indicate that the overexpression of DACH1 can successfully inhibit cellular proliferation and migration of lung cancer cells through the downregulation of PRX 3.

### Clinical significance of DACH1 protein expression in lung adenocarcinoma

In order to study the clinical relevance of DACH1 expression and lung cancer development, this study enrolled 36 patients diagnosed with lung adenocarcinoma ranged from 45 to 74 years, and the mean age at the time of surgery was 58.0 years. Of these patients, 21 were men (77 %) and 15 were women (23 %). Based on the ACCP classification [24], 8 patients were at stage I, 11 were at stage II, and 17 were at stage IIIa. The relative expression of DACH1 in resected lung samples were determined by WB, IHC, and RT-PCR assays (Fig. 1). We next analyzed the correlation between the levels of cellular DACH1 protein and the clinic-pathological parameters of the patients. As shown in Table 1, lower DACH1 expression significantly correlated with tumor diameter (*P = 0.043) and tumor invasion (*P = 0.045). By contrast, no significant association was found between DACH1 downregulation and age, gender, nodal status, or degree of differentiation. These results suggest that DACH1 have physiological function in lung adenocarcinoma proliferation and progression.

**Table 1:** Relationship between DACH1 expression and clinical pathologic parameters

Variables	*N* = 36	DACH1 expression		*p* value
		high (*n* = 19)	low (*n* = 17)	
Age (years)				0.676
≤50	14	8	6	
>50	22	11	11	
Gender				0.535
Male	21	12	9	
Female	15	7	8	
Tumor diameter (cm)				0.043 *
≤2	19	7	12	
>2	17	12	5	
Degree of differentiation				0.194
Poor	15	6	9	
Well to moderate	21	13	8	
Tumor invasion (T)				0.045*
T1/T2	32	15	17	
T3	4	4	0	
Nodal status (N)				0.923
N0	13	7	6	
N+	23	12	11	

*Statistically significant (*p* < 0.05)

## Discussion

In the past 100 years, lung cancer has been transformed from a rare disease into a serious global problem [1, 4]. Although significant advances have been made with conventional therapies, lung cancer still remains the leading cause of cancer-related mortality among both men and women [1, 4, 5], the fact of which calls for improvements in the diagnosis and treatment of the disease. Although the EGFR tyrosine kinase inhibitors gefitinib and erlotinib have revolutionized the treatment of lung cancers, only patients with activating mutations of the EGFR gene respond to the therapy [9]. Also, patients rapidly develop drug resistance during the course of therapy [10], the fact of which highlights the importance of finding out novel therapeutic targets in treating lung cancer.

The *DACH1* gene is a member of the RDGN that regulates retinal cell fate determination [11]. Recently, DACH1 was shown to suppress the growth and invasion of tumor cells in a variety of researches [12–15, 28]. However, the biological function of DACH1 in lung cancer is still not well understood. In this study, we have identified reduced DACH1 expression in lung adenocarcinoma tissues and cells compared to normal tissues and cells, respectively. As a transcription factor, DACH1 likely inhibits tumor growth and progression via transcriptional regulation of target gene expression. Indeed, our in vitro experiment confirmed the growth inhibitory function of DACH1 when overexpressed in lung cancer cells. Furthermore, DACH1 overexpression disrupted cell cycle progression. Consistently, several recent studies reported tumor suppressor function in breast cancer and prostate cancer, and exhibited cell cycle regulatory function [13, 15].

Our study also determined that DACH1 significantly downregulates PRX3 gene expression, which is overexpressed in both of the two lung cancer cells employed in this study. Moreover, we observed a negative correlation between DACH1 and PRX3 expression in clinical lung adenocarcinoma samples and cultured cancer cells. When compared to wild-type lung cancer cells, DACH1 overexpression in LTEP-α-2 and A549 cells blocks cell cycle progression and thus negatively influence tumor cell growth and invasion, while this alteration can be significantly prevented by the co-infection of PRX3. Recently, Zhou et al. demonstrated that DACH1 may inhibit cell cycle progression by competing with FOXM1 for promoter occupation and transcriptional regulation of several cell cycle-related genes [11]. It has been reported that FOXM1 can bind to the PRX3 promoter region containing a FOXM1-binding site [29, 30]. Sung Song et al’s study demonstrated that FOXM1 can transcriptionally activate PRX3 and the stem cell marker CD133, maintaining the stemness in colon cancer stem cells (CSCs) by promoting mitochondrial function [31]. Considering our experimental results and previous publications, it can be concluded that the inhibitory role of DACH1 in the proliferation and invasion of lung adenocarcinoma should be ascribed to the downregulation of its transcriptional target, Prx3, by competing with FOXM1 for promoter binding.

However, Jae-Woong Lee et al’s study demonstrated opposite roles of DACH1 associated with the regulation of the cell cycle machinery and expression of reprogramming factors in myeloid cells [28]. Therefore, it seems that the effect of DACH1 associated with cell fate determination and tumorigenesis has to be differentially considered in different types of tumors. Moreover, this discrepancy suggests the complexity of gene regulation in cancer tissues. We speculate that such gene regulation maybe context dependent, and DACH1 may positively or negatively regulate gene expression through binding to different co-factors [11].

Besides, our study is the first study to statistically reveal that low expression of DACH1 significantly correlated with tumor diameter and invasion in 36 patients diagnosed with lung adenocarcinoma tumor. Thus, downregulation of DACH1 in lung cancer tissues might be an adverse prognostic factor, which should be extensively evaluated in further studies. We will further validate our findings in a larger population in future studies.

In conclusion, the present study demonstrates that DACH1 acts as a potential tumor suppressor in lung cancer tissues and cells through the downregulation of PRX3 expression. Downregulation of DACH1 suggests unfavorable prognosis of lung cancers. All these results indicate that PRX3 might be a novel and ideal target for targeting therapy, which merits further evaluation in the clinic.

## Electronic supplementary material

Below is the link to the electronic supplementary material.

Supplementary Table 1 (DOCX 16 kb)
