# Supplementary material for: DACH1 inhibits the proliferation and invasion of lung adenocarcinoma through the downregulation of peroxiredoxin 3
Source: Tumour Biol. 2016 Jan 25;37(7):9781–8. doi: 10.1007/s13277-016-4811-x (PMC4990600; doi:10.1007/s13277-016-4811-x)
Supplement: Supplementary file 1 — (DOCX 16 kb) [file 13277_2016_4811_MOESM1_ESM.docx]

**Supplementary Table 1: Oligonucleotide primer sequence used in this study**

| **oligonucleotide** | **primer sequences** |
| --- | --- |
| **DACH1** | Forward 5'- CCCTCTACAATGACTGCACCA-3’  Reverse 5'- GCGGCATGATGTGAGAGTTCT-3’ |
| **PRX3** | Forward 5'- GAGACTACGGTGTGCTGTTAGA -3’  Reverse 5'- GTTGACGCTCAAATGCTTGATG -3’ |
